#  Simvastatin induces breast cancer cell death through oxidative stress up-regulating miR-140-5p

**DOI:** 10.18632/aging.101974

**Published:** 2019-05-28

**Authors:** Fuliang Bai, Ze Yu, Xin Gao, Jiawei Gong, Lizhi Fan, Feifei Liu

**Affiliations:** 1 Lubin Environmental Protection Technology (Shanghai) Co., Ltd, Shanghai, China; 2 College of Life Science, Northeast Forestry University, Harbin, China; 3 Department of the Second General Surgery, Jixi Mine Hospital of Heilongjiang, Jixi, China; 4 Department of Geratology, The First Hospital of Harbin in Heilongjiang, Harbin, China; 5 Department of Medical Records, Hongqi Hospital Affiliated to Mudanjiang Medical College, Mudanjiang City, China; * Equal contribution

**Keywords:** Simvastatin, MDA-MB-231, miR-140-5p, NRF1, SLC2A1

## Abstract

Statins, a class of hyperlipidemic drugs, are widely used cholesterol lowering drugs that selectively inhibit 3-hydroxy-3-methylglutaryl CoA reductase, which is the rate-limiting enzyme in cholesterol biosynthesis, leading to decreasing of cholesterol biosynthesis. Statins exert anti-tumoral effects on various cancer, including breast cancer. However, the molecular mechanisms for the actions were not fully elucidated. The purpose of this study was to elucidate the effects of statins on proliferation and apoptosis in the ER-negative breast cancer cell line MDA-MB-231. Our results showed that simvastatin increased the expression of miR-140-5p in a dose dependent manner via activating transcription factor NRF1, reduced cell proliferation and induced apoptosis, and we also found that SLC2A1 was a new target of miR-140-5p. In conclusion, data in this study shed light on the potential anti-tumoral effects of simvastatin in breast cancer and presents a highly promising therapeutic option, using drug and miRNA for combined treating cancers.

## Introduction

Breast cancer is the most common cancer among women all over the world [1,2]. Approximately 15% of breast tumors are triple negative breast cancers (TNBC). TNBC have the poorest survival outcome of all breast cancer subtypes [3]. This is due to its high propensity for metastatic progression and resistance to endocrine therapy [4,5].

Statins have been widely used for inhibiting HMG-CoA reductase (HMGCR), which is regarded as the rate-limiting enzyme to catalyze the important step in mevalonate pathway [6–8]. Some meta-analyses suggested that long-term statin reduces the risk of cancers, including breast cancer [9–12]. In the case of breast cancer, the use of simvastatin is associated with a reduced risk of stage I-III breast cancer [8,10,13]. Preclinical studies have showed that statins participated in apoptosis program and regulation of cell proliferation in breast cancer cells. Simvastatin also exhibits antitumor activity in a variety of cancers including lung cancer and gastric cancer [14–16]. Despite this knowledge, in order to improve and increase the therapeutic effect of simvastatin on cancer, a more complete understanding of simvastatin induced apoptosis is needed.

The contribution of simvastatin to the breast cancer is known as HMGCR inhibitor [17,18]. The mechanisms that simvastatin induced apoptosis in cancer cells including Akt and NF-kB downregulation, nitric oxide and generation of ROS upregulation [17–20]. It was reported that statins inhibited cancer progression by regulating miRNA levels [21–24]. For example, Lovastatin could reduce cell proliferation by upregulating miR-33b expression, impairing c-myc expression and function in medulloblastoma cells [24].

Here, we first demonstrated that simvastatin could up-regulate miR-140-5p in triple negative breast cancer cell line MDA-MB-231 via activating ROS-induced transcription factor NRF1 expression. Our results suggested that simvastatin broke the balance of oxidative stress in MDA-MB-231 cells, resulted in the accumulation of ROS. Subsequently, ROS triggered the increase of NRF1, and NRF1 as a transcription factor bound to the promoter of miR-140 to induce miR-140 expression. Finally, we confirmed that simvastatin combining with miR-140-5p showed the best anticancer effect compared with simvastatin or miR-140-5p treatment alone. Our researches identify a novel pleiotropic anti -cancer effects of simvastatin.

## RESULTS

### Basal expression of cholesterol biosynthesis genes was up-regulated in breast cancer tissue

Simvastatin restrains cholesterol production through inhibiting the rate-limiting enzyme of mevalonate pathway (Supplementary Figure 1A). To explore the significant differences of cholesterol biosynthesis genes in breast normal tissue and breast cancer tissue, we used the Metabolic gEne RApid Visualizer website [25]. Heatmap representations of the differential expression of cholesterol associated transcripts in breast cancer tissue and control breast normal tissue revealed that mevalonate pathway related genes were obviously up-regulated in the tumor (Supplementary Figure 1B). Analysis of the survival curve revealed that the higher mevalonate pathway associated genes (HMGCR, HMGCS1 and INSIG2) levels were correlated with lower survival times for the breast cancer patients [26] (Supplementary Figure 1C). Finally, at the protein level, IHC results showed that the master mevalonate pathway genes, HMGCR and HMGCS1 were also overexpressed in breast cancer tissue [27] (Supplementary Figure 1D). Therefore, these clues strongly indicated that breast cancer had a high dependency on mevalonate pathway.

### Simvastatin-induced MDA-MB-231 cell cytotoxicity

**Figure 1:**
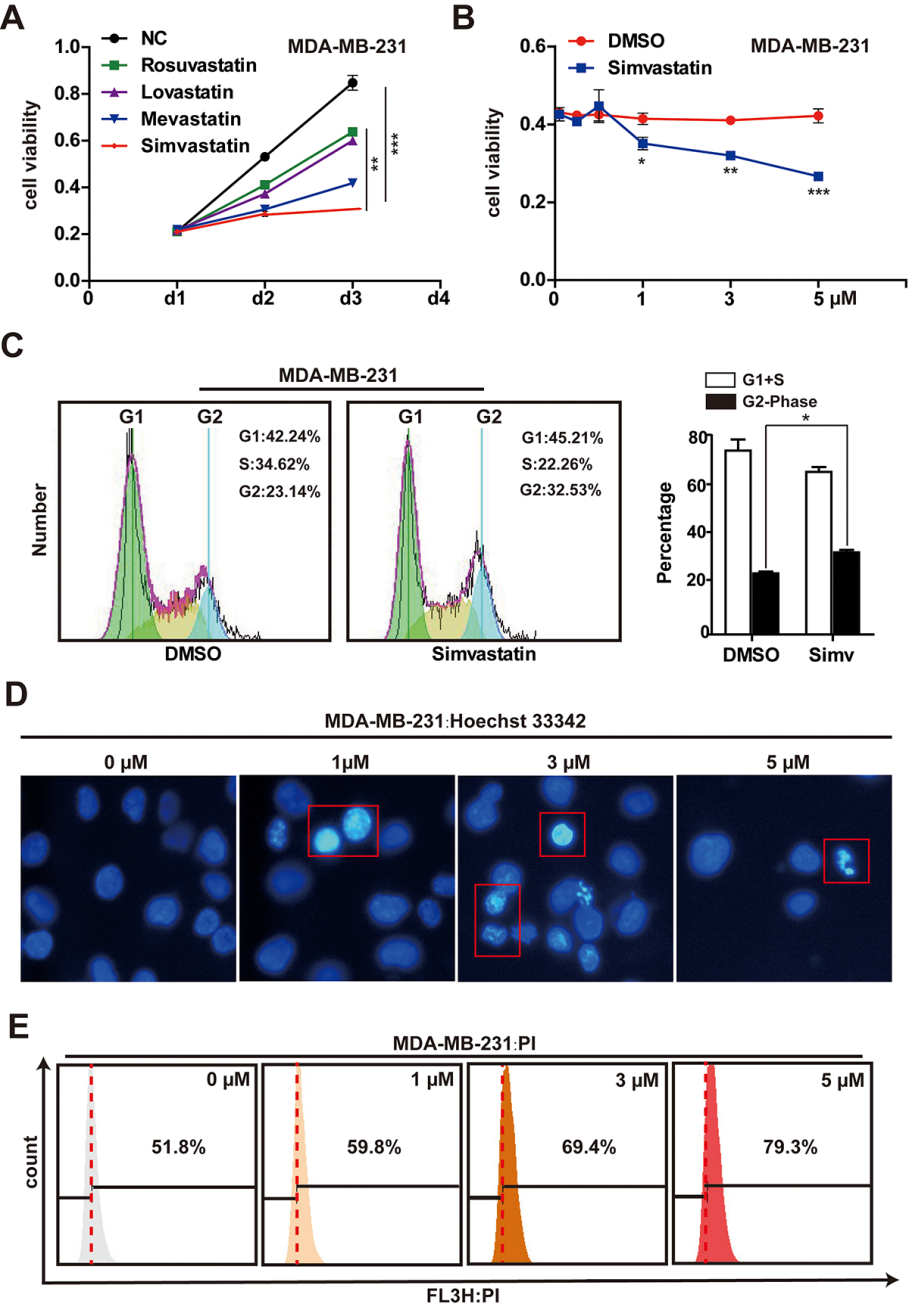
**Effects of simvastatin on cell proliferation and cell death in MDA-MB-231 cells.** (**A**) MDA-MB-231 cells were treated with 1µM rosuvastatin, lovastatin, mevastatin or simvastatin for 24h and 48h. (**B**) MDA-MB-231 cells were treated with varying concentrations (1-5µM) of simvastatin for a period of 48h. All the cell viability (cell proliferation) assays were analyzed by the CCK-8 assay. (**C**) the effects of simvastatin on the cell cycle were measured by flow cytometry with PI staining. (**D**) Representative photomicrography of treated MDA-MB-231 cells with varying doses simvastatin showing nuclei fragmentation. (**E**) MDA-MB-231 were treated with various doses of simvastatin for 48h. Cell death was determined by PI FACS analyses. The percentage of necrotic/apoptosis cells (PI positive) were moved to the right quadrant (Relative to 0µM). The p-values were calculated using standard Student t-tests. Error bars represent mean± SEM of three individual experiments. *** P ≤ 0.001, ** P ≤ 0.01, * P ≤ 0.05.

**Figure 2:**
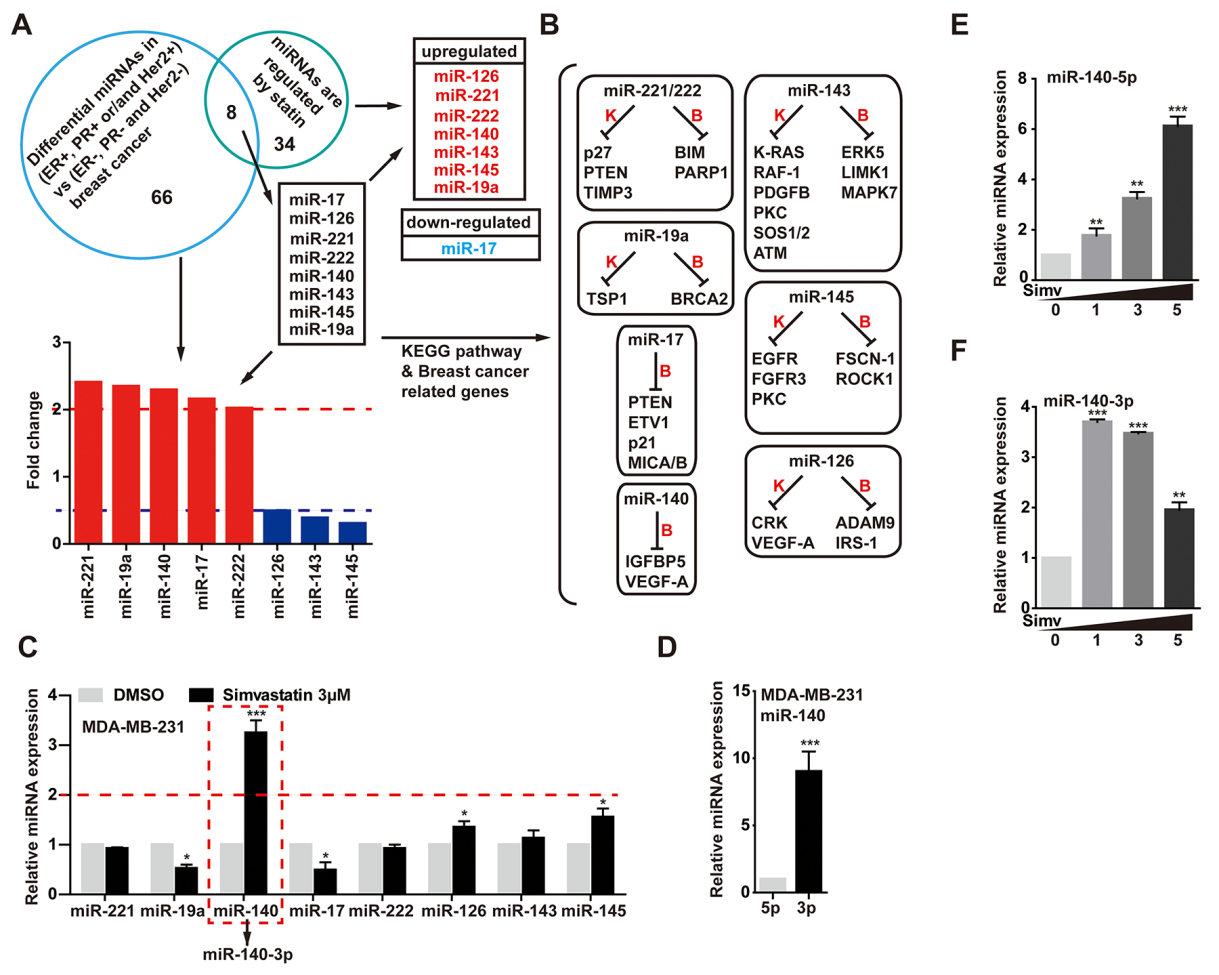
**Simvastatin upregulated miR-140-5p expression.** (**A**) Venn diagram containing miRNAs that were found to be significantly altered in triple negative tumors (ER-, PR- and Her2-) compared with other breast tumors (ER+ and/or PR+ and/or Her2+) and were regulated by statins. (**B**) KEGG pathway showed targeted genes of the 8 different miRNAs came from A. (**C**) qPCR analysis of the 8 different miRNAs expression in MDA-MB-231 cells treated with 3µM simvastatin compared with negative control DMSO for 24h. (**D**) The relative miRNA expression levels of miR-140-5p and miR-140-3p in MDA-MB-231 cells. (**E, F**) The expression levels of miR-140-5p and miR-140-3p were detected by qPCR in MDA-MB-231 cells treated with simvastatin(1-5µM) for 24h. All miRNAs expression was normalized to snRNA U6 housekeeping gene. The p-values were calculated using standard Student t-tests. Error bars represent mean±SEM of three individual experiments. *** P ≤ 0.001, ** P ≤ 0.01.

We assessed the effectiveness of rosuvastatin, lovastatin, mevastatin or simvastatin to induce cytotoxicity in MDA-MB-231 cells. The results showed that MDA-MB-231 cells were most sensitive to the toxicity of simvastatin (Fig, 1A, Supplementary Figure 2A). Therefore, we have been using simvastatin as the studied drug in the subsequent experiments. MDA-MB-231 cells were treated with simvastatin at different concentrations (1-5µM) for 48h. The cell viability (the number of viable cells) was determined using the CCK-8 assay. With the increase of drug concentration, the number of viable cells decreased significantly, suggesting that simvastatin inhibited cell proliferation in a dose-dependent manner (Fig. 1B). In addition, the increase of tumor suppressor gene p21 and p27 was seen in the MDA-MB-231 cells treated with simvastatin (Supplementary Figure 2B). Moreover, cell cycle analysis indicated that simvastatin (3µM) increased the G2 phase of MDA-MB-231 cells (Fig. 1C). At the same time, to study the direct effect of simvastatin on cell death in the MDA-MB-231 cells, we treated MDA-MB-231 cells with simvastatin at various doses, As shown in Fig. 1D, simvastatin significantly caused fragmentation of cell nuclei and induced cell death in a concentration-dependent fashion (Fig. 1E). Furthermore, simvastatin treatment also significantly inhibited the invasion of MDA-MB-231 cells (FSupplementary Figure 2C). These results suggested that inhibition of mevalonate pathway by simvastatin can effectively delay the progression of breast cancer.

### Simvastatin increased miR-140-5p expression in MDA-MB-231 cells

In our experiment, in order to find a simvastatin-regulated microRNA. We first screened the difference miRNAs of TNBC (N=132) tumors compared with other breast tumors (ER + and/or PR + and/or Her2 +, N = 32) [28]. Analysis of these miRNA expression revealed 74 differentially expressed miRNAs, these altered miRNAs possibly were involved in cell proliferation, epithelial mesenchymal transition and deterioration in triple negative tumors. Among the most significant altered miRNAs, we need to narrow down the selection. Second, we found 42 miRNAs that were regulated by statins from Clinical Trials and *in vivo* Studies from Mohajeri's research [29]. Intersected the two results, we discovered 8 different miRNAs. These miRNAs may contribute to the aggressive phenotype of TNBC cancers and promote tumor progression, and at the same time, the levels of these miRNAs were regulated by statins (Fig. 2A). As shown in Fig. 2B, previous studies had reported that miR-143, miR-126, miR-145 and miR-140 play the role as tumor suppressors in breast cancer, but miR-221/222, miR-17 and miR-19a function as oncogenes in breast cancer. Next, we validated the expression levels of these microRNAs in MDA-MB-231 cells treated with simvastatin, a qPCR assay showed that simvastatin induced miR-140, miR-126 and miR-145 expression, while miR-17 and miR-19a were down-regulated in MDA-MB-231 cells (Fig. 2C).

Exhilaratingly, there was a significant change in miR-140 level upon simvastatin treatment in MDA-MB-231 cells. Interestingly, further studies found that simvastatin-induced miR-140 was miR-140-3p, while the screened miR-140 in GSE86278 database was miR-140-5p. To investigate the miR-140 expression in breast cancer cell, we examined the expression levels of miR-140-3p and 5p in MDA-MB-231 cells. As the data displayed, miR-140-5p expression was decreased at least eightfold in MDA-MB-231 cells reduced as compared with the miR-140-3p (Fig. 2D). The YM500v2 miRNA database showed that miR-140-3p was dominant in most human tissues compared with miR-140-5p [30] (Supplementary Figure 3A-C). Although the level of miR-140-3p was much higher than miR-140-5p, simvastatin induced miR-140-5p up-regulation in a dose-dependent manner, while miR-140-3p was down-regulated in the case of increased simvastatin concentration (1-5µM) (Fig. 2E, F). The over expression of miR-140-5p significantly reduced cell growth, while miR-140-3p did not work (Supplementary Figure 3D). These data suggested a possible tumor suppressor activity of miR-140-5p induced by simvastatin in triple negative breast cancer cell line.

### Simvastatin induced pre-miR-140 expression via up-regulating NRF1

**Figure 3:**
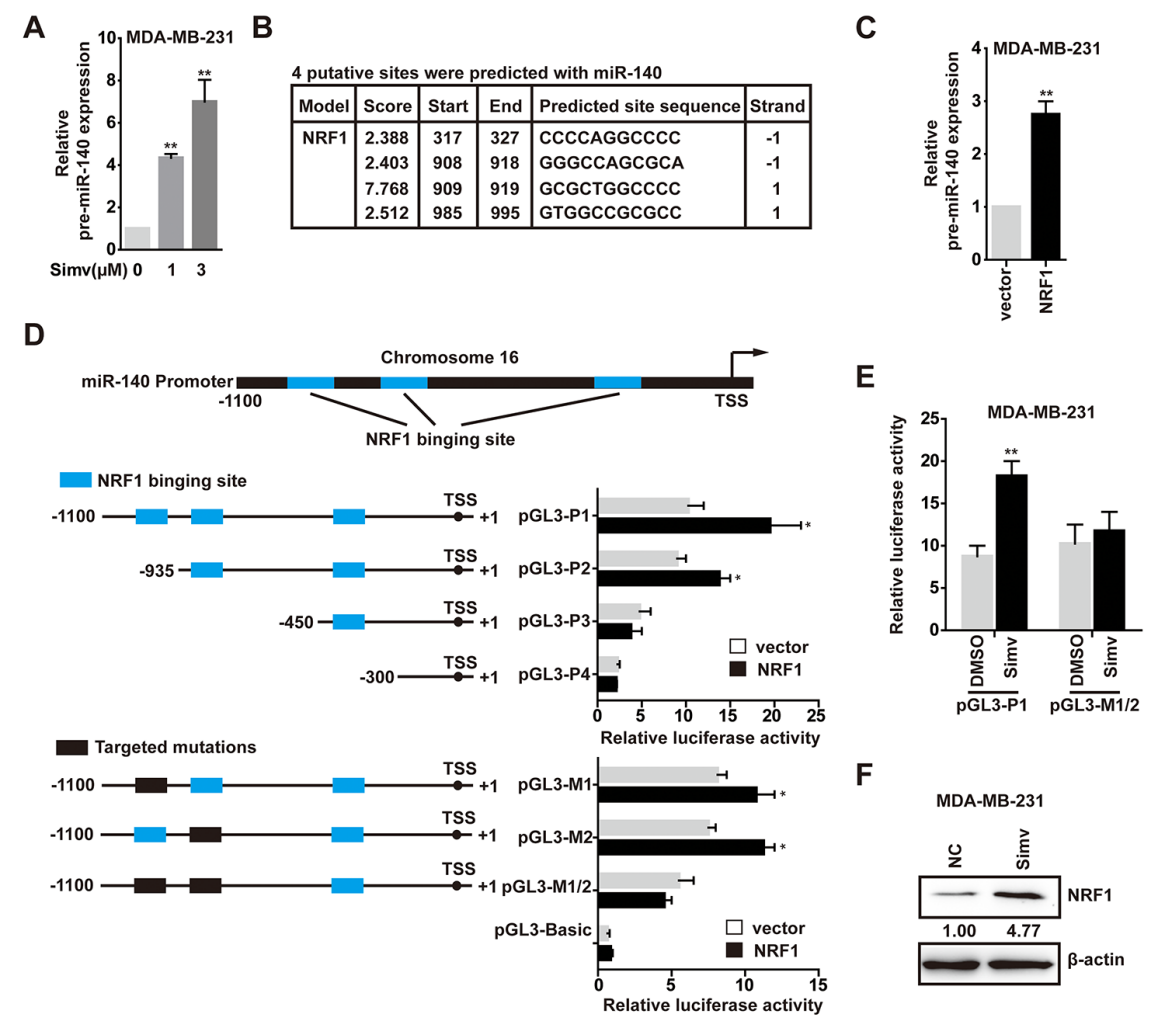
**NRF1 bound to and activated the pre-miR-140 promoter.** (**A**) The expression levels of pre-miR-140 was detected by qPCR in MDA-MB-231 cells treated with simvastatin(1-3µM) for 24h. (**B**) The location of NRF1-binding sites in the pre-miR-140 proximal promoter region was predicted by the JASPAR CORE database. (**C**) The relative miRNA expression levels of pre-miR-140 in MDA-MB-231 cells transfected with NRF1 over-expressing plasmid compared with empty plasmid. (**D**) Sequential deletion and mutation analyses identified NRF1-responsive regions in the pre-miR-140 proximal promoter region. pGL3-P2, pGL3-P3 and pGL3-P4 represented the deletion, and pGL3-M1, pGL3-M2 and pGL3-M1/2 represented the mutation. Serially truncated and mutated pre-miR-140 promoter vectors were co-transfected with NRF1 over-expressing plasmid or empty plasmid into MDA-MB-231 cells, and the relative luciferase activities were determined. (**E**) Effect of simvastatin on pre-miR-140 promoter driven luciferase activity. MDA-MB-231 cells were transfected with pGL3-P1 or pGL3-M1/2 plasmids, along with 3µM simvastatin or DMSO. (**F**) MDA-MB-231 cells were cultured and treated with 3µM simvastatin or NC(DMSO) for 24h and NRF1 protein was measured by Western blot. β-actin served as a control. The p-values were calculated using standard Student t-tests. Error bars represent mean±SEM of three individual experiments. ** P ≤ 0.01.

Considering both of miR-140-3p and miR-140-5p were upregulated at the low concentration of simvastatin, so we speculated that simvastatin could induce pre-miR-140 expression. As shown in Fig. 3A, treatment with simvastatin enhanced the pre-miRNA level of miR-140 in a dose-dependent manner. We found several potential binding sites for NRF1 are present in the pre-miR-140 proximal promoter through searching the JASPAR CORE database (Fig. 3B).

We next examined whether NRF1 was involved in pre-miR-140 expression. First, we certified that the level of pre-miR-140 was increased in MDA-MB-231 cells following treatment with over-expression NRF1 (Fig. 3C). Second, to determine the role of NRF1 in the transcriptional regulation of pre-miR-140, we used the luciferase reporter assay to demonstrate that augmentation of NRF1 stimulated the pre-miR-140 promoter in HEK293T cells. To identify which site was functionally required for NRF1-regulated pre-miR-140 promoter activation, a deletion analysis of the pre-miR-140 promoter in HEK293T cells identified the NRF1-responsive region in -450 to -1100. Deletion of the region containing only the -985 to -995 site also caused a significant decrease in pre-miR-140 promoter activity upon NRF1 over-expression (Fig. 3D). A significant reduction in pre-miR-140 promoter activity was observed when the -908 to -919 and -985 to -995 sites were individually or jointly mutated (Fig. 3D). These results demonstrated that both the -908 to -919 and -985 to -995 sites were essential for NRF1-regulated pre-miR-140 promoter activity. Consistent with the luciferase data, results revealed that simvastatin effectively stimulated pre-miR-140 promoter (Fig. 3E). It indicated that simvastatin increased the transcription of pre-miR-140 via promoting NRF1 activity or up-regulating NRF1. Further experiments confirmed that simvastatin could promote NRF1 expression (Fig. 3F). Taken together, these findings suggested that NRF1 was essential for the expression of pre-miR-140, and simvastatin increased NRF1-regulated pre-miR-140 level in a dose-dependent manner.

### Simvastatin induced oxidative stress and DNA damage

**Figure 4:**
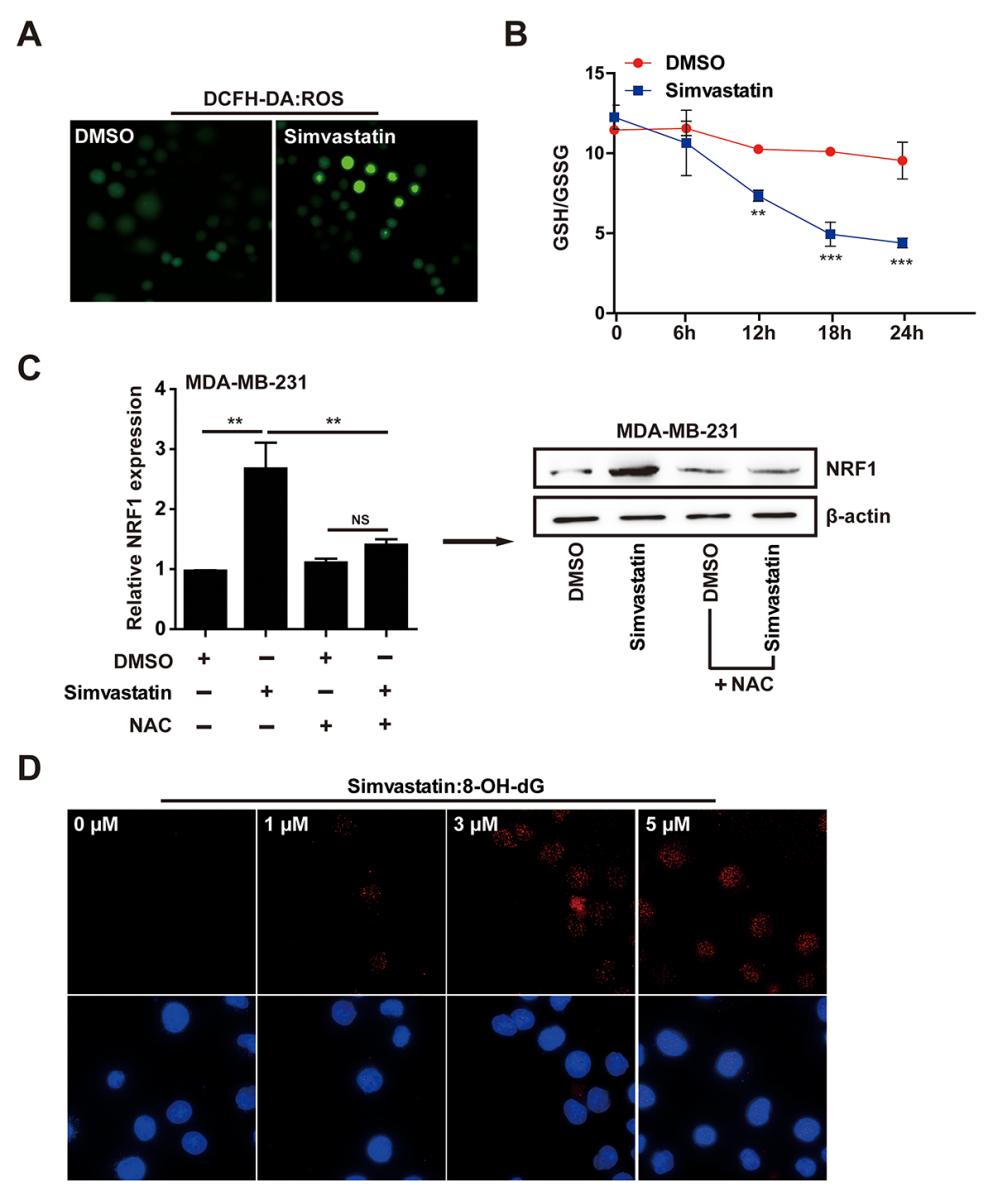
**Simvastatin induced oxidative stress.** (**A**) The ROS level showed effects in MDA-MB-231 cells with simvastatin or DMSO treatment for 24h as determined by fluorescence intensity of DCFH-DA (ROS probe). (**B**) simvastatin induced ROS elevation accompanied by a continuous decline of GSH/GSSG ratio in MDA-MB-231 cells within 24 hours. (**C**) The effect of simvastatin and NAC on NRF1 expression as detected by qPCR and western blot. (**D**) DNA oxidative damage marker 8-OH-dG was measured by ICC/IF in MDA-MB-231 cells treated with simvastatin(1-5µM) for 24h. The p-values were calculated using standard Student t-tests. Error bars represent mean±SEM of three individual experiments. *** P ≤ 0.001, ** P ≤ 0.01.

Nuclear NRF1 binds to antioxidant response elements (ARE) and activates the transcription of antioxidant and anti-inflammation genes, so NRF1 is a ROS-induced oxidative stress sensitive transcription factors [31,32]. To explore the mechanism of action by which simvastatin upregulated NRF1, we investigated the accumulation of ROS in MDA-MB-231 cells treated with simvastatin by using fluorescent probe DCFH-DA. Simvastatin significantly increased cellular ROS levels (fluorescence intensity) in MDA-MB-231 cells (Fig. 4A). At the same time, as shown in Fig. 4B, accompanied by simvastatin-treated time increase, a decrease of ratio (GSH/GSSG) was found in MDA-MB-231 cells. To show the direct role of ROS in enhancing the expression of NRF1, we co-treated the simvastatin treated MDA-MB-231 cells with NAC to show a direct effect on NRF1 expression and consequent regulation of pre-miR-140. As shown in the data (Fig. 4C), NAC could significantly suppress the up-regulation of NRF1 expression induced by simvastatin and the consequent increase in pre-miR-140 level (Supplementary Figure 3E).

The above results indicated that the increase of NRF1 induced by simvastatin was due to the accumulation of ROS. 8-OH-dG, as a product of DNA damage induced by ROS, is a biomarker of oxidative DNA damage. Simvastatin can induce the production of 8-OH-dG in a dose-dependent manner, indicating that increased ROS caused severe cell cytotoxicity (Fig. 4D). These results revealed that simvastatin -ROS-NRF1 axis was contributed to the increased of miR-140.

### SLC2A1 is a novel direct target of miR-140-5p

**Figure 5:**
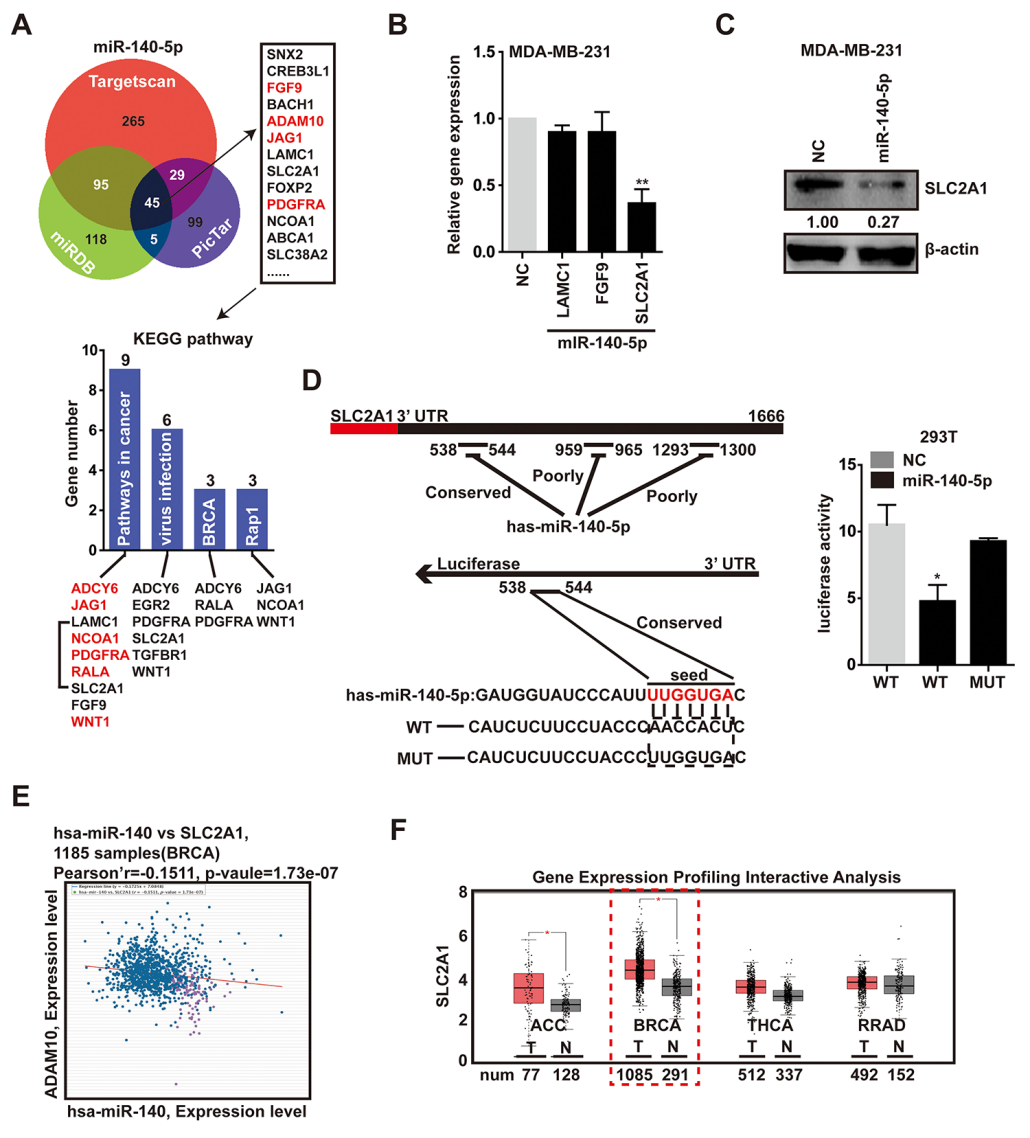
**SLC2A1 is the direct target of miR-140-5p.** (**A**) Venn diagram containing genes that were predicted to be the targets of miR-140-5p, and the KEGG pathway analysis of the 45 putative genes which were in the intersection. Red represented reported targets of miR-140-5p. (**B**) The expression levels of LAMC1, SLC2A1 and FGF9 in MDA-MB-231 cells transfected with miR-140-5p mimic compared with NC mimic for 48h. (**C**) The transfection of miR-140-5p in MDA-MB-231 cells decreased the SLC2A1 protein levels, as shown by western blot. (**D**) Left, luciferase-SLC2A1 3′-UTR constructs. 3 putative miR-140-5p binding sequences existed in the 3′-UTR of SLC2A1 mRNA, one was conservative, and the other two were poorly-conservative. miR-140-5p seed mutated sequences were generated in the binding site. Right, luciferase reporter assay in HEK293 cells transfected with NC, miR-140-5p or miR-140-5p-mut 3′-UTR. Firefly luciferase served as an internal control. (**E**) Expression patterns of miR-140-5p with SLC2A1 exhibited a negative correlation. (**F**) The expression level of miR-140-5p was decreased in BRCA compared with normal tissues. This difference was also reflected in ACC, but there is no significant disparity in THCA and RRAD. The p-values were calculated using standard Student t-tests. Error bars represent mean±SEM of three individual experiments. * P ≤ 0.05.

To explore the mechanism by which the down-modulation of miR-140-5p impacts breast cancer programs, we screened the potential target genes of miR-140-5p through integration of several databases, including targetscan [33], miRDB [34] and PicTar [35], and this analysis showed that there were 45 putative genes objectives of miR-140-5p in all three databases. Subsequent KEGG pathway analysis of the 45 genes showed that important pathways were associated with cancer progression, viral infection, BRCA and Rap1 pathway (Fig. 5A). Of these, the cancer progression contained the greatest number of genes. Remarkably, red-labeled genes were miR-140-5p target genes that had been reported, and the remaining genes, including LAMC1, SLC2A1 and FGF9 were not previously reported (Fig. 5A). Among these genes, the basal expression level of SLC2A1 is the highest not only in MDA-MB-231 cells, but also in breast tumor (Supplementary Figure 4A).

**Figure 6:**
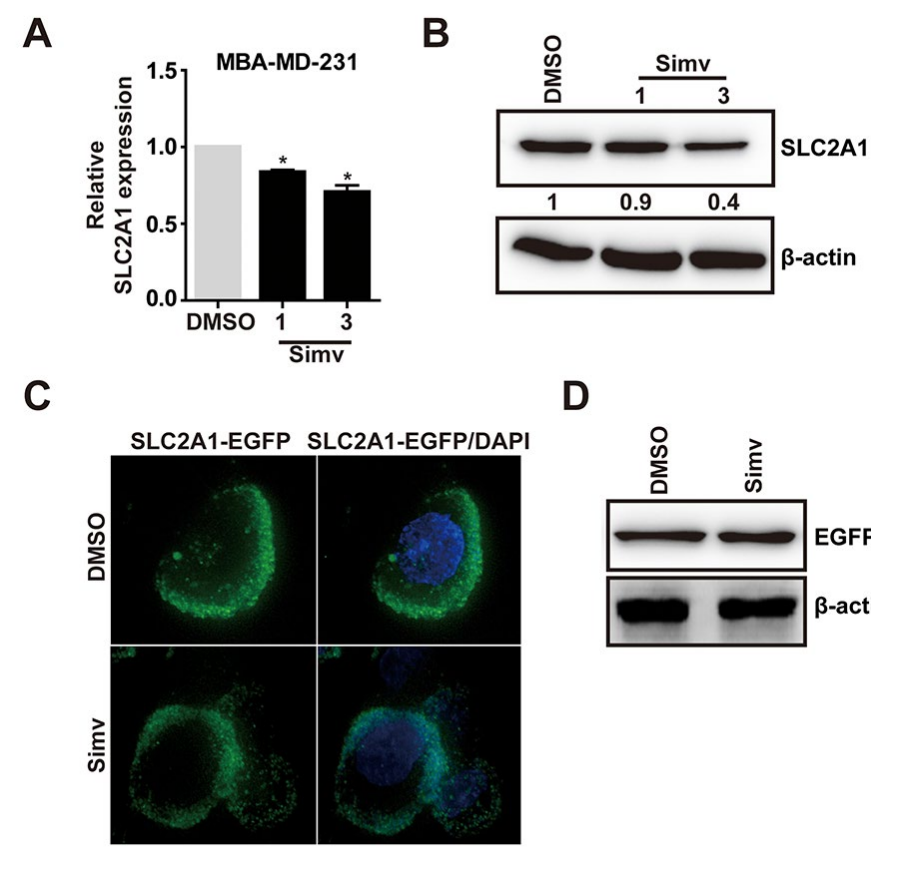
**Simvastatin inhibited SLC2A1 expression.** (**A, B**) MDA-MB-231 cells were treated with either NC(DMSO) or various concentrations of simvastatin(1-3µM) for 24h. The mRNA and protein expression of SLC2A1 were examined by qPCR and western blot, respectively. (**C**) Effect of simvastatin on the localization of SLC2A1. MDA-MB-231 cells were transfected with EGFP-SLC2A1 co-expression plasmid for 48h, then the cells were treated with either DMSO or simvastatin for 24h and observed with a Delta Vision Imaging Workstation. (**D**) Effect of simvastatin on the exogenous EGFP-SLC2A1 through western blot analysis. The p-values were calculated using standard Student t-tests. Error bars represent mean±SEM of three individual experiments. * P ≤ 0.05.

To confirm the direct inhibition of predicted genes by miR-140-5p, we evaluated their mRNA level after the exogenous expression of the miR-140-5p in MDA-MB-231 cells. The data displayed the mRNA level of SLC2A1 was reduced in cells transfected with miR-140-5p mimics in a concentration-dependent way (Fig. 5B, Supplementary Figure 4B). And the protein expression level of SLC2A1 was also decreased in miR-140-5p transfected cells (Fig. 5C). But there was no significant difference in the other two genes (Fig. 6B). Next, we transfected HEK293T cells with luciferase reporter containing a reporter plasmid fused with the conservative 3′UTR miR-140-5p binding sequence of SLC2A1, and exogenous miR-140-5p mimic. This result showed an approximate 50% decrease in luciferase activity, whereas the mutant seed region was not decreased (Fig. 5D). In addition, the mRNA expression level of SLC2A1 achieved by ChIPBase was inversely correlated with those of miR-140-5p in BRCA samples [36] (Fig. 5E). To further determine the correlation between miR-140-5p and SLC2A1, we screened the potential microRNAs targeting SLC2A1 expression via targetscan and miRDB, and the bioinformatic prediction indicated that just miR-140-5p and miR-152-3p were predicted targeting SLC2A1 (Supplementary Figure 4C).

Meanwhile, we analyzed the gene expression profiling data of SLC2A1 from GEPIA [37], and found SLC2A1 was significantly up-regulated in breast tumor compared with normal tissue (Fig. 5F). In conclusion, these data confirmed that SLC2A1 was an exact target gene of miR-140-5p.

### Simvastatin inhibited the expression of SLC2A1

In previous studies, we found that simvastatin upregulated miR-140-5p and SLC2A1 was the downstream target gene of miR-140-5p. Next, we need to determine whether simvastatin could inhibit SLC2A1 in MDA-MB-231 cells. Q-PCR and Western blot analysis showed that simvastatin suppressed SLC2A1 in a dose dependent way (Fig. 6A, B). However, simvastatin did not decrease the exogenous SLC2A1 expression and membrane localization in MDA-MB-231 cells (Fig. 6C, D). These outcomes showed that simvastatin only decreased the expression of endogenous SLC2A1.

### Combination of simvastatin and miR-140-5p led to promote cell death

**Figure 7:**
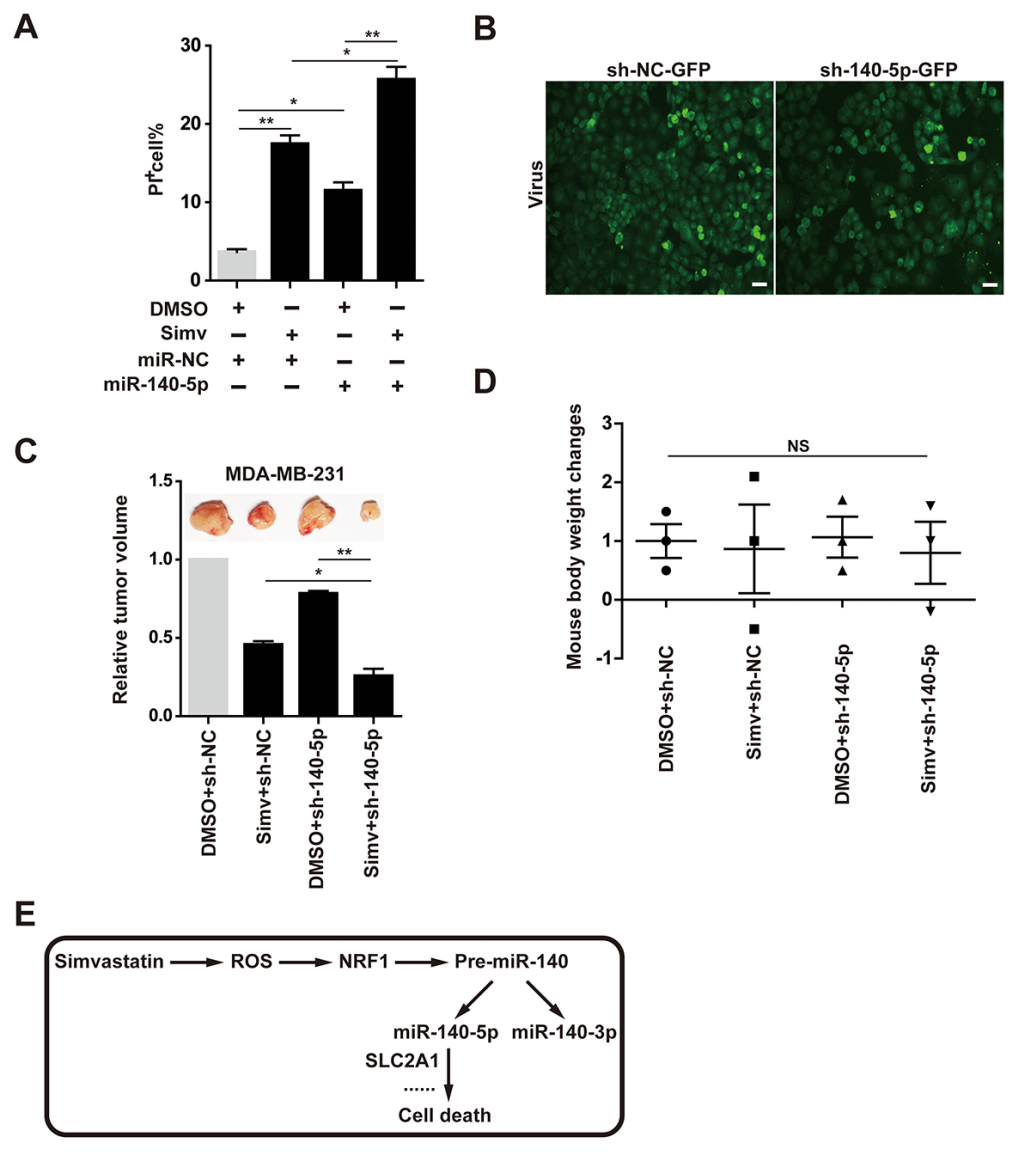
**Combination of simvastatin and miR-140-5p promoted cell death *in vitro* and *in vivo*.** (**A**) cell death induced by the four groups treatment (DMSO+ NC, simvastatin+ NC, DMSO+ miR-140-5P and simvastatin+ miR-140-5P) was measured by counting PI positive cell number. (**B**) lentiviral vector-infected MDA-MB-231cells and miR-140-5P vector-infected cells fluorescence detection. (**C**) Tumor volume detection in the four groups treatment. (**D**) Body weight change in the four groups of xenografted mice. (**E)** Schematic diagram of the mechanism that simvastatin-induced cell death by up-regulating miR-140-5P. The p-values were calculated using standard Student t-tests. Error bars represent mean± SEM of three individual experiments. ** P ≤ 0.01, * P ≤ 0.05.

Induction of cell death by simvastatin and miR-140-5p was examined by counting PI positive cell number in MDA-MB-231 cells. The results showed that both simvastatin and miR-140-5p alone increase cell death, and combination of miR-140-5p transfection with simvastatin led to a significantly increase of cell death compared with the them alone (Fig. 7A). miR-140-5p or negative control designed as a short hairpin RNA (shRNA) was brought into MDA-MB-231 cells by a lentivirus vector, which has an EGFP Reporter (Fig. 7B), then we injected MDA-MB-231 cells treated with miR-140-5p or NC stably expressing lentivirus into the immune-deficient mice. After 6 weeks, we killed these mice and examined the volume of tumor derived from these xenografted mice. These results indicated that tumor volume in the group combining of miR-140-5p overexpressing cells and simvastatin feeding were less than those of the other three groups, showing the best anti-cancer effect (Fig. 7C). At the same time, we detected the body weight of the xenografted mice administrated with DMSO and simvastatin, and did not find significant changes in all groups (Fig. 7D).

## DISCUSSION

In the present study, simvastatin showed the best anticancer effect compared with other types of statins. This result suggested that breast cancer may be more sensitive to simvastatin than other types of statins, such as rosuvastatin, lovastatin, and mevastatin. Thus, we suspected that simvastatin had pleiotropic effects as it may interact with diverse targets, such as miRNAs. Therefore, we further explored the regulatory relationship between simvastatin and miRNAs in accordance with this idea, and found that simvastatin-ROS-NRF1 -miR-140-5p axis effectively inhibited breast cancer cells (Fig. 7E).

Accumulating evidences have demonstrated that statins could reduce the risk of multiple cancers, including breast cancer [9,10,13]. *In vitro* and *in vivo* studies have also shown that statins played important roles in depressing proliferation and inducing apoptosis of breast cancer [38,39]. Thus, statins have been regarded as potential anti-cancer therapeutics agent.

Previously, researchers have found that simvastatin significantly increased the levels of ROS and malondialdehyde (MDA) in a dose-dependent manner for 48h [20,40]. MDA, a natural production of lipid oxidation, is used to evaluate the levels of oxidative stress and lipid oxidation. Their results showed MDA degree exhibited a significant dose-dependent on the concentration of simvastatin40. At the same time, another research investigated the role of simvastatin-induced apoptosis in A549 cells by ROS accumulation [41]. On the other hand, in MCF-7 cells, simvastatin induced intracellular ROS production, increased cytochrome c protein expression and caspase-3 activity [42].

Recently, data implicated that ROS could activate transcription factors NRF1, NRF2 and CREB, increase production of ROS, which played crucial role in cell proliferation and migration in breast cancer through increasing genomic instability and activating aforesaid redox sensitive transcription factors [43]. Importantly, ROS can function as a signal molecule to trigger the downstream target genes of transcription factors NRF1 and NRF2 which were involved in the progression of breast cancer [32].

MicroRNAs are a group of non-coding RNAs molecules, the dysregulation of which play key role in tumor proliferation, metastasis and angiogenesis [44,45]. A previous research demonstrated that miR-140-5p was frequently downregulated in breast cancer cells, and miR-140-5p could suppress cell proliferation via directly targeting VEGF-A in MDA-MB-231 and MCF-7 cells [46]. Here focusing on the current study, our data showed that miR-140-5p inhibited cell proliferation, and the results *in vitro* were consistent with the cell experiments. Notably, in this study we found that miR-140-5p regulating by simvastatin could suppress tumor growth compared with the NC.

Importantly, we found that SLC2A1 was a novel target gene of miR-140-5p. There is an inverse relationship between the expression level of miR-140-5p and SLC2A1 in breast cancer patient samples. Solute carrier family 2 (SLC2A1), also named glucose transporter 1 (GLUT1), is a major glucose transporter in mammalian cells [47,48]. Cancer cells are known to reprogram metabolism to support rapid cell proliferation, for their increased energy demands, the significant features of this regulative metabolism are elevated glucose uptake [49]. Therefore, as an important member of the GLUT family, GLUT1 is abnormally elevated in many solid tumors, including breast cancer (Fig. 6F), and it is considered a potential therapeutic target. A plenty of studies showed that GLUT1 is involved in the progression of cancer cell [50–52], and silencing Glut1 might enhance anticancer effect of chemotherapeutic agents in triple-negative breast cancer (TNBC) cell lines [53].

In conclusion, the present study demonstrated that miR-140-5p enhanced simvastatin-induced cytotoxicity towards MDA-MB-231 cells through inhibition of the SLC2A1 gene, which was the direct target of miR-140-5p. The combination treatment with simvastatin and miR-140-5p potentiated their apoptotic activity over that used with either method alone in MDA-MB-231 cells. To our knowledge, this is the first study to show significant association between the simvastatin and tumor suppressor miR-140-5p. First, we identified that simvastatin is a effective anticancer drug, which significantly increases ROS production and upregulated NRF1 and miR-140 expression. Second, we demonstrated that NRF1 contributed to the expression of the ARE-dependent miR-140. Last, we demonstrated that ROS activated NRF1 and promoted transcription of the downstream genes of NRF1. Taken together, these results indicated that simvastatin up-regulated miR-140-5p through activating the ROS-NRF1-miR-140 axis in MDA-MB-231 cells. Findings in the current study exhibited a novel simvastatin-mediated regulatory network for breast cancer, which may provide a novel therapeutic target in the treatment of breast cancer.

## MATERIALS AND METHODS

### Cell culture

The human breast cancer cell line MDA-MB-231 were obtained from the American Type Culture Collection (Chicago, IL, USA) and cultured RPMI-1640 medium supplemented with 10% fetal bovine serum (FBS), 100 U/ml of penicillin and streptomycin (Sigma, St. Louis, MO, USA), 2mM L-glutamine. The cultured cells were maintained in a 5% CO2 concentration at 37 °C.

### Cell viability assay

Cell viability was examined using the cell counting Kit-8 (CCK-8, Beyotime, Shanghai, China) assay according to the instructions from the manufacturer. MDA-MB-231 cells were placed in a 96-well plate at a density of 5000 cells/well. Cells were allowed to adhere for 24 h at 37 °C and then treated with various statins (rosuvastatin, lovastatin, mevastatin or simvastatin). The supernatants were removed and replaced with 100μl of fresh medium containing 10μl of CCK-8 solution and the cells were incubated for 2 h at 37 °C in the dark. Immediately after the incubation, the optical density of each well at 450 nm (OD value) was measured with a microplate Reader (sunrise TECAN, JAPAN). Cell viability was expressed as percentage absorbance of cells treated with inhibitors compared with the percentage absorbance of untreated cells.

### Cell cycle analysis by flow cytometry

After treated with simvastatin for 48 hours, MDA-MB-231 cells were digested, collected and washed with PBS twice. After being fixed in 70% ethanol at 4°C for 1h, the cells were resuspended in a staining solution of 50 μg/mL propidium iodide (PI) (Vazyme Biotec, Nanjing, China), 1 mg/mL RNase A, and 0.1% Triton X-100 in PBS for 1h. The stained cells were then analyzed with a flow cytometer (BD Accuri C6).

### Morphological observation of cell death by fluorescence microscopy

MDA-MB-231 cells were treated with various concentrations of simvastatin for 24h. Afterwards, the nucleus of these cells was stained with Hoechst 33342. Fluorescence microscopy analysis and apoptotic morphology such as nuclei fragmentation was performed with a Zeiss Axiostart Fluorescence Microscope.

### Measurement of cell death by flow cytometry

Cell death was measured by flow cytometry using PI stain for simvastatin cytotoxic experiments. MDA-MB-231 cells, treated with different doses of simvastatin, were kept under stress conditions for 24h before the cell death assay. These cells were harvested and washed once in cold PBS, and then stained with PI for 30 min at room temperature in the dark. After staining, the cells were analyzed by flow cytometry using 488 nm excitation. The percentage of death cells corresponds to PI-positive cells. All samples were analyzed in a flow cytometry (BD C6 Biosciences, San Jose, CA, USA).

### Cell invasion assay

Cell invasion was tested using a transwell assay system (Corning, USA). The simvastatin-treated MDA-MB-231 cells were seeded with serum-free RPMI-1640 medium and plated into the upper layer polycarbonate membrane filter, RPMI-1640 medium with 10% FBS was added to the bottom chambers. After 72 h, the cells that crossed upper layer polycarbonate to the bottom chambers were fixed with 4% PFA, stained with 0.05% crystal violet and counted.

### Immunofluorescence analysis of 8-OH-dG

The levels of 8-OH-dG were measured using immunofluorescence. MDA-MB-231 cells, treated with different doses of simvastatin, were fixed by 4% PFA for 30min, the culture was washed with PBS 3 times. After permeabilization, 50μL 8-OH-dG antibody (Abcam, ab48508) was added at 1:200 dilution 37°C for 30 min. After several washes, cells were incubated with Alexa Fluor 594 donkey anti-goat IgG secondary antibody (Life Technologies, cat#A-11058).

### Measurement of intracellular ROS levels

Intracellular ROS generation was assessed using the peroxide-sensitive fluorescent probe 2′,7′-dichlorofluorescin diacetate (DCFH-DA; Beyotime, China). in accordance with the instructions. MDA-MB-231 cells were incubated with the dye of DCFH-DA, which was attenuated with serum-free DMEM at a proportion of 1:1000, for 30 min at 37 °C in the dark, washed twice with PBS and then detected the generation of intracellular ROS by fluorescence microscope for magnifications 40×, and the fluorescence analysis was performed using ImageJ software.

### Determination of GSH and GSSG

The intra-cellular GSH and GSSG level was measured by GSH and GSSG Assay Kit (S0053) from Beyotime Biotechnology. MDA-MB-231 cells were treated with 3µM simvastatin for different hours, collected and then lyzed by releasing buffer on ice. According to the protocols of manufacturer, the standard curve of the absorbance to GSH and GSSG concentrations was measured. Then We determined the GSH and GSSG concentration using microplate reader at 412 nm.

### Plasmid construction

Over-expression NRF1 vector construction: First, we amplified the coding sequence of NRF1 gene from MDA-MB-231 cells by PCR. The amplified sequence was then cloned into a pcDNA3.1+ expression vector (Invitrogen) using BamHI and XhoI endonucleases (Takara). The control vector was pcDNA3.1+ (empty carrier).

Over-expression SLC2A1 vector construction: We amplified the coding sequence of SLC2A1 gene from MDA-MB-231 cells by PCR. The amplified sequence was then cloned into a pEGFP-N2 expression vector (Clontech) using XhoI and BamHI endonucleases (Takara). The control vector was pEGFP-N2 (empty carrier). All the primers were seen in Supplementary material 2.1-2.2.

### Dual-Luciferase reporter assay

NRF1 and miR-140 promoter: Luciferase reporter vector contained the miR-140 promoter region with a deletion of NRF1 regulatory element ranging from -1100 to +1, -935 to +1, -450 to +1, -300 to +1 relative to the transcription start site (TSS). These sequences were copied from genomic DNA of MDA-MB-231 cells and inserted into the pGL3-Basic vector (Promega, USA) using KpnI and HindIII endonucleases, respectively. Three site mutant vectors (pGL3-MT1, pGL3-MT2, and pGL3-MT1/2) were generated using overlap-PCR method. To analyze promoter activity, the MDA-MB-231 cells were co-transfected with NRF1- overexpressed constructs and miR-140 promoter vectors or pRL-TK vectors (control) in MDA-MB-231 cells.

miR-140-5p and the 3'-UTR of SLC2A1: The 3'-UTR of SLC2A1 containing the miR-140-5p binding sites or mutated sequences were cloned into the pMIR-report luciferase reporter vector (Promega) using MluI and SpeI.

The reconstructive luciferase vectors were named pMIR-SLC2A1-3UTR-WT and pMIR-SLC2A1-3UTR-MUT. The MDA-MB-231 cells were co-transfected with the reporter vectors and miR-140-5p mimics or negative control (NC). After 48 hours, Firefly and Renilla luciferase activities were measured by the GloMax-20/20 Luminometer from Promega. And the relative promoter activity was normalized by endogenous Renilla luciferase activity. Primer sequences were shown in Supplementary material 2.3-2.10.

### Real-time PCR

Total RNA was extracted using the TRIzol RNA isolation system (Takara, Dalian, China) according to the manufacturer's instructions. mRNA was reversely transcribed using random primers, while miRNA was reversely transcribed with oligod(T)-ambion and the total RNA required 3'Poly(A) tail addition treatment.

Real-time PCR was performed using SYBR Green Master (Cat#04913914001, Roche) in a Light-Cycler 480 System (Roche, Basel, Switzerland) was used for determination of mRNA and miRNA levels. β-actin was used as the internal normalization control for mRNA and pre-miRNA, and snRNA U6 was used as control for mature miRNA. The primer sequences used are listed in Supplementary material 2.11-2.29. The relative gene expression levels were quantified by normalization to endogenous β-actin or U6 expression levels, which were calculated by the 2-Δ ΔC (t) method.

### Western blot analysis

The cell lysate was prepared using RIPA buffer containing protease inhibitors, phosphatase inhibitors, and dithiothreitol. Protein extracts from MDA-MB-231 cells were prepared and protein concentration was measured using a BCA protein assay kit (Beyotime, China). Western blot analyses were performed with the use of specific antibody for NRF1 (Cell Signaling, Cat# 46743), EGFP (Beyotime, AG281), SLC2A1 (abcam, ab190163) and β-actin (A5441) from Sigma. Goat anti-rabbit IgG-horseradish peroxidase (HRP) was from Cell Signaling Technology. Relative quantification of protein levels was determined by measuring the intensity of the protein bands with the use of ImageJ software.

### Lentiviral transduction

All viruses were packaged using the third generation lentivector system (Invitrogen) and expressed in HEK293T cells. The supernatant, which contains the virus, was collected at 48h and concentrated using 10-kDa amicon Ultra-15 centrifugal filter units (Millipore, USA). Overexpression of sh-NC or sh-140-5p was achieved by cloning using the pLu-Puro-Indu-shRNA virus vector, respectively. (Systems Biosciences, USA).

### Tumor xenograft assay

Male immune-deficient (NPG) mice (5 weeks old) were purchased from the Vital River Laboratory Animal Technology (Beijing). For xenografts, 1 million cells suspended in 100μL of PBS was injected subcutaneously into the NPG mice (n = 5 per group). MDA-MB-231 cells infected with sh-NC or sh-140-5p retroviral were injected into the flanks of NPG mice. These xenograft mice drank the water with DMSO and simvastatin, respectively and separated into four groups: sh-NC+ DMSO, sh-NC+ simvastatin, sh-140-5p+ DMSO and sh-140-5p+ simvastatin. After 4 weeks, all mice were killed, and tumor volume were measured. Tumor volume was calculated as follows: Tumor volume = (length x width2)/2. All xenograft animal experiments were supervised by the committees for the ethical review of research at the Harbin Medical Sciences University.

### Bioinformatics analyses

All bioinformatics analysis methods and websites were shown in Supplementary material 2.

### Data analysis

All the bars or symbols in the graph represent the means ± standard deviation error from at least three independent experiments with similar results. The results were analyzed by the Student's t-test and in all analysis, * P < 0.05, ** P < 0.01 and *** P < 0.001 were considered statistically significant.

## SUPPLEMENTARY MATERIAL

Supplementary Methods 2

Supplementary Figures

## References

[1] Kumar P, Aggarwal R. An overview of triple-negative breast cancer. Arch Gynecol Obstet. 2016; 11 10 3198 3219:247–69. 10.1007/s00404-015-3859-y 26341644

[2] Marotti JD, de Abreu FB, Wells WA, Tsongalis GJ. Triple-Negative Breast Cancer: Next-Generation Sequencing for Target Identification. Am J Pathol. 2017; 187:2133–38. 10.1016/j.ajpath.2017.05.018 28734944

[3] Sharma P. Biology and Management of Patients With Triple-Negative Breast Cancer. Oncologist. 2016; 21:1050–62. 10.1634/theoncologist.2016-0067 27401886 PMC5016071

[4] Harbeck N, Gnant M. Breast cancer. Lancet. 2017; 389:1134–50. 10.1016/S0140-6736(16)31891-8 27865536

[5] Liedtke C, Rody A. New treatment strategies for patients with triple-negative breast cancer. Curr Opin Obstet Gynecol. 2015; 27:77–84. 10.1097/GCO.0000000000000137 25502428

[6] Sirtori CR. The pharmacology of statins. Pharmacol Res. 2014; 88:3–11. 10.1016/j.phrs.2014.03.002 24657242

[7] Bedi O, Dhawan V, Sharma PL, Kumar P. Pleiotropic effects of statins: new therapeutic targets in drug design. Naunyn Schmiedebergs Arch Pharmacol. 2016; 389:695–712. 10.1007/s00210-016-1252-4 27146293

[8] Ahern TP, Lash TL, Damkier P, Christiansen PM, Cronin-Fenton DP. Statins and breast cancer prognosis: evidence and opportunities. Lancet Oncol. 2014; 15:e461–68. 10.1016/S1470-2045(14)70119-6 25186049 PMC4167822

[9] Islam MM, Yang HC, Nguyen PA, Poly TN, Huang CW, Kekade S, Khalfan AM, Debnath T, Li YJ, Abdul SS. Exploring association between statin use and breast cancer risk: an updated meta-analysis. Arch Gynecol Obstet. 2017; 296:1043–53. 10.1007/s00404-017-4533-3 28940025

[10] Wu QJ, Tu C, Li YY, Zhu J, Qian KQ, Li WJ, Wu L. Statin use and breast cancer survival and risk: a systematic review and meta-analysis. Oncotarget. 2015; 6:42988–3004. 10.18632/oncotarget.5557 26472026 PMC4767486

[11] Tan P, Zhang C, Wei SY, Tang Z, Gao L, Yang L, Wei Q. Effect of statins type on incident prostate cancer risk: a meta-analysis and systematic review. Asian J Androl. 2017; 19:666–71. 10.4103/1008-682X.190327 27924788 PMC5676426

[12] Singh S, Singh PP, Singh AG, Murad MH, Sanchez W. Statins are associated with a reduced risk of hepatocellular cancer: a systematic review and meta-analysis. Gastroenterology. 2013; 144:323–32. 10.1053/j.gastro.2012.10.005 23063971

[13] Shai A, Rennert HS, Lavie O, Ballan-Haj M, Bitterman A, Steiner M, Keren S, Rennert G. Statins, aspirin and risk of venous thromboembolic events in breast cancer patients. J Thromb Thrombolysis. 2014; 38:32–38. 10.1007/s11239-013-1015-8 24154915

[14] Ahmed TA, Hayslip J, Leggas M. Pharmacokinetics of high-dose simvastatin in refractory and relapsed chronic lymphocytic leukemia patients. Cancer Chemother Pharmacol. 2013; 72:1369–74. 10.1007/s00280-013-2326-3 24162379

[15] Han JY, Lee SH, Yoo NJ, Hyung LS, Moon YJ, Yun T, Kim HT, Lee JS. A randomized phase II study of gefitinib plus simvastatin versus gefitinib alone in previously treated patients with advanced non-small cell lung cancer. Clin Cancer Res. 2011; 17:1553–60. 10.1158/1078-0432.CCR-10-2525 21411446

[16] Kim ST, Kang JH, Lee J, Park SH, Park JO, Park YS, Lim HY, Hwang IG, Lee SC, Park KW, Lee HR, Kang WK. Simvastatin plus capecitabine-cisplatin versus placebo plus capecitabine-cisplatin in patients with previously untreated advanced gastric cancer: a double-blind randomised phase 3 study. Eur J Cancer. 2014; 50:2822–30. 10.1016/j.ejca.2014.08.005 25218337

[17] Kotamraju S, Williams CL, Kalyanaraman B. Statin-induced breast cancer cell death: role of inducible nitric oxide and arginase-dependent pathways. Cancer Res. 2007; 67:7386–94. 10.1158/0008-5472.CAN-07-0993 17671209

[18] Sánchez CA, Rodríguez E, Varela E, Zapata E, Páez A, Massó FA, Montaño LF, Lóopez-Marure R. Statin-induced inhibition of MCF-7 breast cancer cell proliferation is related to cell cycle arrest and apoptotic and necrotic cell death mediated by an enhanced oxidative stress. Cancer Invest. 2008; 26:698–707. 10.1080/07357900701874658 18608208

[19] Hwang KE, Na KS, Park DS, Choi KH, Kim BR, Shim H, Jeong ET, Kim HR. Apoptotic induction by simvastatin in human lung cancer A549 cells via Akt signaling dependent down-regulation of survivin. Invest New Drugs. 2011; 29:945–52. 10.1007/s10637-010-9450-2 20464445

[20] Ahn KS, Sethi G, Aggarwal BB. Simvastatin potentiates TNF-alpha-induced apoptosis through the down-regulation of NF-kappaB-dependent antiapoptotic gene products: role of IkappaBalpha kinase and TGF-beta-activated kinase-1. J Immunol. 2007; 178:2507–16. 10.4049/jimmunol.178.4.2507 17277159

[21] Karlic H, Thaler R, Gerner C, Grunt T, Proestling K, Haider F, Varga F. Inhibition of the mevalonate pathway affects epigenetic regulation in cancer cells. Cancer Genet. 2015; 208:241–52. 10.1016/j.cancergen.2015.03.008 25978957 PMC4503872

[22] Peng X, Li W, Yuan L, Mehta RG, Kopelovich L, McCormick DL. Inhibition of proliferation and induction of autophagy by atorvastatin in PC3 prostate cancer cells correlate with downregulation of Bcl2 and upregulation of miR-182 and p21. PLoS One. 2013; 8:e70442. 10.1371/journal.pone.0070442 23936432 PMC3731278

[23] Docrat TF, Nagiah S, Krishnan A, Naidoo DB, Chuturgoon AA. Atorvastatin induces MicroRNA-145 expression in HEPG2 cells via regulation of the PI3K/AKT signalling pathway. Chem Biol Interact. 2018; 287:32–40. 10.1016/j.cbi.2018.04.005 29630879

[24] Takwi AA, Li Y, Becker Buscaglia LE, Zhang J, Choudhury S, Park AK, Liu M, Young KH, Park WY, Martin RC, Li Y. A statin-regulated microRNA represses human c-Myc expression and function. EMBO Mol Med. 2012; 4:896–909. 10.1002/emmm.201101045 22887866 PMC3491823

[25] Shaul YD, Yuan B, Thiru P, Nutter-Upham A, McCallum S, Lanzkron C, Bell GW, Sabatini DM. MERAV: a tool for comparing gene expression across human tissues and cell types. Nucleic Acids Res. 2016; 44:D560–66. 10.1093/nar/gkv1337 26626150 PMC4702927

[26] Shi J, Dong B, Zhou P, Guan W, Peng Y. Functional network analysis of gene-phenotype connectivity associated with temozolomide. Oncotarget. 2017; 8:87554–67. 10.18632/oncotarget.20848 29152101 PMC5675653

[27] Pontén F, Schwenk JM, Asplund A, Edqvist PH. The Human Protein Atlas as a proteomic resource for biomarker discovery. J Intern Med. 2011; 270:428–46. 10.1111/j.1365-2796.2011.02427.x 21752111

[28] Romero-Cordoba SL, Rodriguez-Cuevas S, Bautista-Pina V, Maffuz-Aziz A, D’Ippolito E, Cosentino G, Baroni S, Iorio MV, Hidalgo-Miranda A. Loss of function of miR-342-3p results in MCT1 over-expression and contributes to oncogenic metabolic reprogramming in triple negative breast cancer. Sci Rep. 2018; 8:12252. 10.1038/s41598-018-29708-9 30115973 PMC6095912

[29] Mohajeri M, Banach M, Atkin SL, Butler AE, Ruscica M, Watts GF, Sahebkar A. MicroRNAs: Novel Molecular Targets and Response Modulators of Statin Therapy. Trends Pharmacol Sci. 2018; 39:967–81. 10.1016/j.tips.2018.09.005 30249403

[30] Cheng WC, Chung IF, Tsai CF, Huang TS, Chen CY, Wang SC, Chang TY, Sun HJ, Chao JY, Cheng CC, Wu CW, Wang HW. YM500v2: a small RNA sequencing (smRNA-seq) database for human cancer miRNome research. Nucleic Acids Res. 2015; 43:D862–67. 10.1093/nar/gku1156 25398902 PMC4383957

[31] Lacher SE, Alazizi A, Wang X, Bell DA, Pique-Regi R, Luca F, Slattery M. A hypermorphic antioxidant response element is associated with increased MS4A6A expression and Alzheimer’s disease. Redox Biol. 2018; 14:686–93. 10.1016/j.redox.2017.10.018 29179108 PMC5705802

[32] Ohtsuji M, Katsuoka F, Kobayashi A, Aburatani H, Hayes JD, Yamamoto M. Nrf1 and Nrf2 play distinct roles in activation of antioxidant response element-dependent genes. J Biol Chem. 2008; 283:33554–62. 10.1074/jbc.M804597200 18826952 PMC2662273

[33] Agarwal V, Bell GW, Nam JW, Bartel DP. Predicting effective microRNA target sites in mammalian mRNAs. eLife. 2015; 4:e05005. 10.7554/eLife.05005 26267216 PMC4532895

[34] Wong N, Wang X. miRDB: an online resource for microRNA target prediction and functional annotations. Nucleic Acids Res. 2015; 43:D146–52. 10.1093/nar/gku1104 25378301 PMC4383922

[35] Salehi Z, Akrami H. Target genes prediction and functional analysis of microRNAs differentially expressed in gastric cancer stem cells MKN-45. J Cancer Res Ther. 2017; 13:477–83. 28862212 10.4103/0973-1482.213691

[36] Zhou KR, Liu S, Sun WJ, Zheng LL, Zhou H, Yang JH, Qu LH. ChIPBase v2.0: decoding transcriptional regulatory networks of non-coding RNAs and protein-coding genes from ChIP-seq data. Nucleic Acids Res. 2017; 45:D43–50. 10.1093/nar/gkw965 27924033 PMC5210649

[37] Tang Z, Li C, Kang B, Gao G, Li C, Zhang Z. GEPIA: a web server for cancer and normal gene expression profiling and interactive analyses. Nucleic Acids Res. 2017; 45:W98–102. 10.1093/nar/gkx247 28407145 PMC5570223

[38] Gopalan A, Yu W, Sanders BG, Kline K. Simvastatin inhibition of mevalonate pathway induces apoptosis in human breast cancer cells via activation of JNK/CHOP/DR5 signaling pathway. Cancer Lett. 2013; 329:9–16. 10.1016/j.canlet.2012.08.031 22960596

[39] Shen YY, Yuan Y, Du YY, Pan YY. Molecular mechanism underlying the anticancer effect of simvastatin on MDA-MB-231 human breast cancer cells. Mol Med Rep. 2015; 12:623–30. 10.3892/mmr.2015.3411 25738368

[40] Li Y, Fu J, Yuan X, Hu C. Simvastatin inhibits the proliferation of A549 lung cancer cells through oxidative stress and up-regulation of SOD2. Pharmazie. 2014; 69:610–14. 10.1691/ph.2014.4508 25158572

[41] Hwang KE, Kim YS, Hwang YR, Kwon SJ, Park DS, Cha BK, Kim BR, Yoon KH, Jeong ET, Kim HR. Enhanced apoptosis by pemetrexed and simvastatin in malignant mesothelioma and lung cancer cells by reactive oxygen species-dependent mitochondrial dysfunction and Bim induction. Int J Oncol. 2014; 45:1769–77. 10.3892/ijo.2014.2584 25096993

[42] Buranrat B, Suwannaloet W, Naowaboot J. Simvastatin potentiates doxorubicin activity against MCF-7 breast cancer cells. Oncol Lett. 2017; 14:6243–50. 10.3892/ol.2017.6783 29113274 PMC5661424

[43] Okoh V, Deoraj A, Roy D. Estrogen-induced reactive oxygen species-mediated signalings contribute to breast cancer. Biochim Biophys Acta. 2011; 1815:115–33. 10.1016/j.bbcan.2010.10.005 21036202

[44] Hammond SM. An overview of microRNAs. Adv Drug Deliv Rev. 2015; 87:3–14. 10.1016/j.addr.2015.05.001 25979468 PMC4504744

[45] Di Leva G, Garofalo M, Croce CM. MicroRNAs in cancer. Annu Rev Pathol. 2014; 9:287–314. 10.1146/annurev-pathol-012513-104715 24079833 PMC4009396

[46] Lu Y, Qin T, Li J, Wang L, Zhang Q, Jiang Z, Mao J. MicroRNA-140-5p inhibits invasion and angiogenesis through targeting VEGF-A in breast cancer. Cancer Gene Ther. 2017; 24:386–92. 10.1038/cgt.2017.30 28752859 PMC5668497

[47] Szablewski L. Glucose transporters in healthy heart and in cardiac disease. Int J Cardiol. 2017; 230:70–75. 10.1016/j.ijcard.2016.12.083 28034463

[48] Amann T, Hellerbrand C. GLUT1 as a therapeutic target in hepatocellular carcinoma. Expert Opin Ther Targets. 2009; 13:1411–27. 10.1517/14728220903307509 19874261

[49] Tekade RK, Sun X. The Warburg effect and glucose-derived cancer theranostics. Drug Discov Today. 2017; 22:1637–53. 10.1016/j.drudis.2017.08.003 28843632

[50] Brown RS, Goodman TM, Zasadny KR, Greenson JK, Wahl RL. Expression of hexokinase II and Glut-1 in untreated human breast cancer. Nucl Med Biol. 2002; 29:443–53. 10.1016/S0969-8051(02)00288-3 12031879

[51] Martel F, Guedes M, Keating E. Effect of polyphenols on glucose and lactate transport by breast cancer cells. Breast Cancer Res Treat. 2016; 157:1–11. 10.1007/s10549-016-3794-z 27097608

[52] Wang J, Ye C, Chen C, Xiong H, Xie B, Zhou J, Chen Y, Zheng S, Wang L. Glucose transporter GLUT1 expression and clinical outcome in solid tumors: a systematic review and meta-analysis. Oncotarget. 2017; 8:16875–86. 10.18632/oncotarget.15171 28187435 PMC5370007

[53] Lin C, Xu X. YAP1-TEAD1-Glut1 axis dictates the oncogenic phenotypes of breast cancer cells by modulating glycolysis. Biomed Pharmacother. 2017; 95:789–94. 10.1016/j.biopha.2017.08.091 28892790

