# Supplementary material for: Simvastatin induces breast cancer cell death through oxidative stress up-regulating miR-140-5p
Source: Aging (Albany NY). 2019 May 28;11(10):3198–219. doi: 10.18632/aging.101974 (PMC6555469; doi:10.18632/aging.101974)
Supplement: Supplementary Methods 2 [file aging-11-101974-s001.pdf]

## SUPPLEMENTARY MATERIAL

## Supplementary Methods 2

### Primer sequences for *vector construction*:

the italics represent the enzyme digestion site

***1. NRF1 vector construction***
Forward: CGC*GGATCC*ATGGAGGAACACGGAGTGAC (BamHI)
Reverse: CCG*CTCGAG*TCACTGTTCCAATGTCACCACCT (XhoI)

***2. SLC2A1 vector construction***
Forward: CCG*CTCGAG*ATGGAGCCCAGCAGCAAGAAGCT (XhoI)
Reverse: CGC*GGATCC*GTCACTTGGGAATCAGCCCCCAGGG (BamHI)

***3. pGL3-P1 construction***
Forward: CGG*GGTACC*TCCCTCCCAAAGCTCTCATGC (KpnI)

***4. pGL3-P2 construction***
Forward: CGG*GGTACC*GGAGAAACCCCGTCTCTA (KpnI)

***5. pGL3-P3 construction***
Forward: CGG*GGTACC*GGGCAGGTGAAGTCAGGTGTCC (KpnI)

***6. pGL3-P4 construction***
Forward: CGG*GGTACC*TTCACCCTGGTCCACCCATA (KpnI)
pGL3-P1, pGL3-P2, pGL3-P3 and pGL3-P4 shared the reverse primer.
Reverse: CCC*AAGCTT*ACACAGGGCGCGGCCACACCAGAA (HindIII)

***7. pGL3-M1 construction (overlap-PCR)***
Reverse: CAGGCCCCTGTTCCAGGCCCAAT, PCR with the forward of *pGL3-P1*
Forward: GGGGCCTGGGGTGCCCCACTGGGC, PCR with the reverse of *pGL3-P1*

***8. pGL3-M2 construction (overlap-PCR)***
Reverse: ATCCATCCACGGGGCCAGCGCAG, PCR with the forward of *pGL3-P1*
Forward: TTCTGCGCTGGCCCCGTGGATGGAT, PCR with the reverse of *pGL3-P1*

***9. pMIR-SLC2A1-3UTR-WT construction***
Forward: CG*ACGCGT*CACCCAGCTAATCTGTAGG (MulI)
Reverse: CGG*ACTAGT*GAGTTTGCAGGCTCCCACAG (SpeI)

***10. pMIR-SLC2A1-3UTR-MUT construction (overlap-PCR)***
Reverse: GATTAATTTGTCACCAAGGG,
PCR with the forward primer of *pMIR-SLC2A1-3UTR-WT*
Forward: CCCTTGGTGACAAATTAATC,
PCR with the reverse primer of *pMIR-SLC2A1-3UTR-WT*

### Primer for real-time PCR:

*11. LAMC1:* Forward: GGACTCCGCCCGAGGAATA;
Reverse: ACTTGAGACGCACATAGGTGA
*12. SLC2A1:* Forward: GGCCAAGAGTGTGCTAAAGAA;
Reverse: ACAGCGTTGATGCCAGACAG
*13. FGF9:* Forward: GGCCTGGTCAGCATTCGAG;
Reverse: GTATCGCCTTCCAGTGTCCAC
*14. p21:* Forward: CGATGGAACTTCGACTTTGTCA
Reverse: GCACAAGGGTACAAGACAGTG
*15. p27:* Forward: AACGTGCGAGTGTCTAACGG
Reverse: CCCTCTAGGGGTTTGTGATTCT
*16. pre-miR-140:* Forward: TCTCTGTGTCCTGCCAGTGGTTT
Reverse: CGGTATCCTGTCCGTGGTTCTAC
*17. β-actin:* Forward: CTCCATCCTGGCCTCGCTGT

Reverse: GCTGTCACCTTCACCGTTCC

### Primer for mature miRNA detection:

18. *oligod(T)-ambion:*
*GCGAGCACAGAATTAATACGACTCACTATAGGTTTTTTTTTTTT*
19. *miR-221-3p:* Forward: AGCTACATTGTCTGCTGGGTTTC
20. *miR-222-3p:* Forward: AGCTACATCTGGCTACTGGGT
21. *miR-19a-3p:* Forward: TGTGCAAATCTATGCAAAACTGA
22. *miR-140-3p:* Forward: TACCACAGGGTAGAACCACGG
23. *miR-140-5p:* Forward: GATGGTATCCCATTTTGGTGAC
24. *miR-17-5p:* Forward: CAAAGTGCTTACAGTGCAGGTAG
25. *miR-126-3p:* Forward: TCGTACCGTGAGTAATAATGCG
26. *miR-143-3p:* Forward: TGAGATGAAGCACTGTAGCTC
27. *miR-145-5p:* Forward: GTCCAGTTTTCCCAGGAATCCCT
28. *Universal reverse primer (19-27):* GCGAGCACAGAATTAATACGAC
29. *SnRNA U6:* Forward: CGCTTCGGCAGCACATATAC

Reverse: TTCACGAATTTGCGTGTCAT

### Bioinformatics analyses

1. The heatmap analysis of expression levels of cholesterol biosynthesis genes in breast normal tissue and breast cancer tissue. These data came from the Metabolic gEne RApid Visualizer website (MERAV).
(http://merav.wi.mit.edu/SearchByGenes.html)
2. The immunohistochemical analysis of HMGCR and HMGCS1 were obtained from The Human Protein Atlas. (https://www.proteinatlas.org/)
3. The correlation of HMGCR, HMGCS1 and INSIG2 mRNA levels and the survival time was analysed using data from the OncoLnc database.
(http://www.oncolnc.org/)
4. The miRNA expression data regarding TNBC tumors and other breast tumors (ER + and/or PR + and/or Her2 +, N = 32) (GSE86278) was downloaded from the GEO database. (https://www.ncbi.nlm.nih.gov/gds/)
5. KEGG pathway analysis were performed through the online database DAVID. (https://david.ncifcrf.gov/)
6. The high-quality transcription factor binding profile database JASPAR was used to predict the transcription factors which bound to pre-miR-140 promoter.
(http://jaspardev.genereg.net/)
7. Prediction of microRNA targets: targetscan, miRDB and PicTar.
Targetscan: (http://www.targetscan.org/mamm_31/);
miRDB: (http://mirdb.org/)
PicTar: (https://pictar.mdc-berlin.de/)
8. the expression levels of SLC2A1 mRNA achieved by ChIPBase were correlated with those of miR-140-5p in BRCA samples. (http://rna.sysu.edu.cn/chipbase/)
9. The expression levels of SLC2A1 in ACC, BRCA, THCA and RRAD were obtained via the GEPIA website: Gene Expression Profiling Interactive Analysis.
(http://gepia.cancer-pku.cn/)
10. the level of miR-140-3p was compared with miR-140-5p. these results were from the YM500v2 miRNA database.
(http://ngs.ym.edu.tw/ym500v2/index.php)
