# Supplementary material for: Simvastatin induces breast cancer cell death through oxidative stress up-regulating miR-140-5p
Source: Aging (Albany NY). 2019 May 28;11(10):3198–219. doi: 10.18632/aging.101974 (PMC6555469; doi:10.18632/aging.101974)
Supplement: Supplementary Figures [file aging-11-101974-s002.pdf]

Supplementary Figures

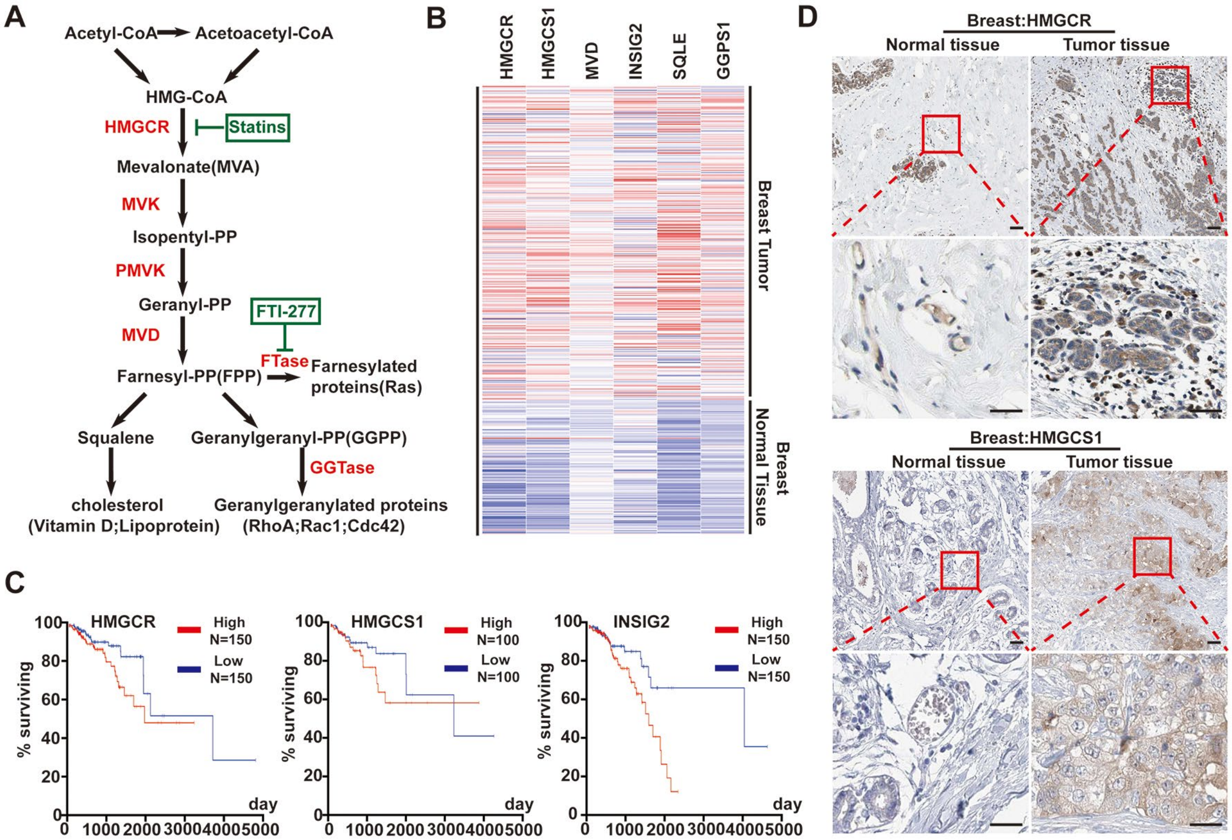

Figure S1. Metabolic transcript screening identifies the mevalonate pathway is a dysregulated metabolism in breast cancer. (**A**) Simplified schematic of the mevalonate pathway. (**B**) The heat map illustrated overexpressed transcripts (HMGCR, HMGCS1, MVD, INSIG2, SQLE and GGPS1) in breast cancer tissues, involved in mevalonate pathway, by analyzing the data from the MERAV database. (**C**) Kaplan–Meier overall survival curves for breast cancer patients, divided into high- and low- HMGCR, HMGCS1 and INSIG2 expression groups. (**D**) IHC staining was performed in breast normal tissue and cancer tissue. Changes in expression of HMGCR and HMGCS1.

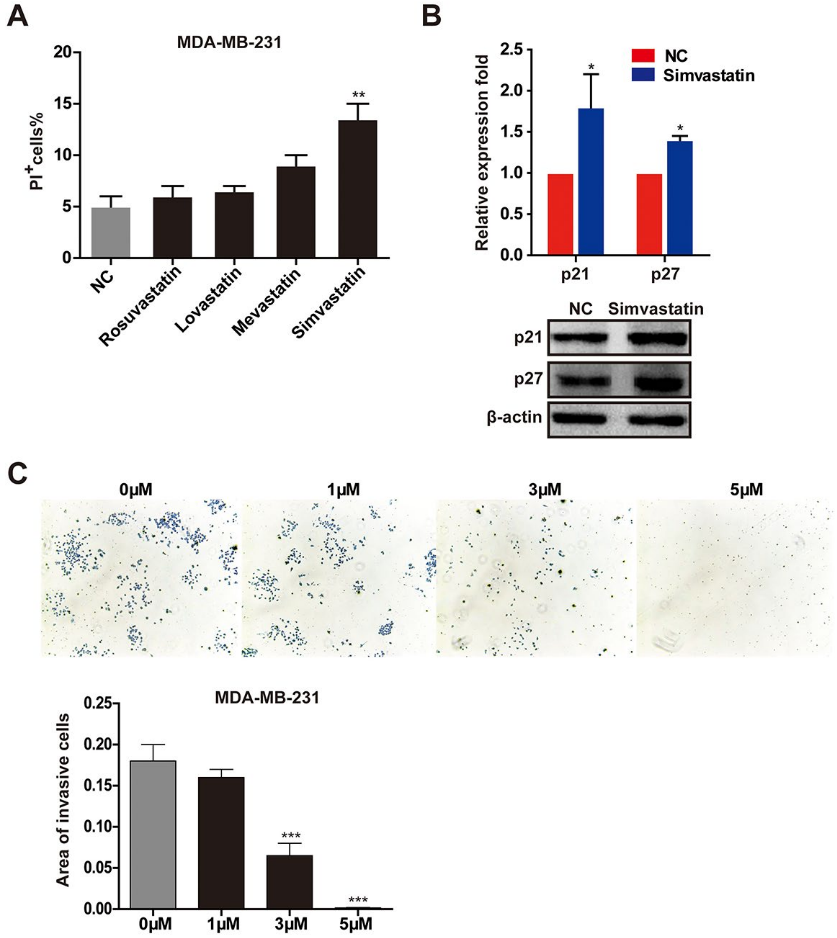

Figure S2. Effects of simvastatin on cancer progression in MDA-MB-231 cells. (**A**) MDA-MB-231 cells were treated with 1μM rosuvastatin, lovastatin, mevastatin or simvastatin for 48h. Cell death was determined by PI FACS analyses. Statistical results showed the percentage of PI +cells. (**B**) MDA-MB-231 cells were treated with 1μM simvastatin or control (DMSO) for 48h. The mRNAs and proteins were collected for qPCR analysis and western blot of tumor suppressor gene p21 and p27, respectively. Actin was used as the control. (**C**) The invasion of MDA-MB-231 cells transfected with various doses (1-5μM) of simvastatin for 24h was determined via Transwell assay. Each individual experiment has been repeated three times. The significance level was *** P ≤ 0.001, ** P ≤ 0.01, * P ≤ 0.05.

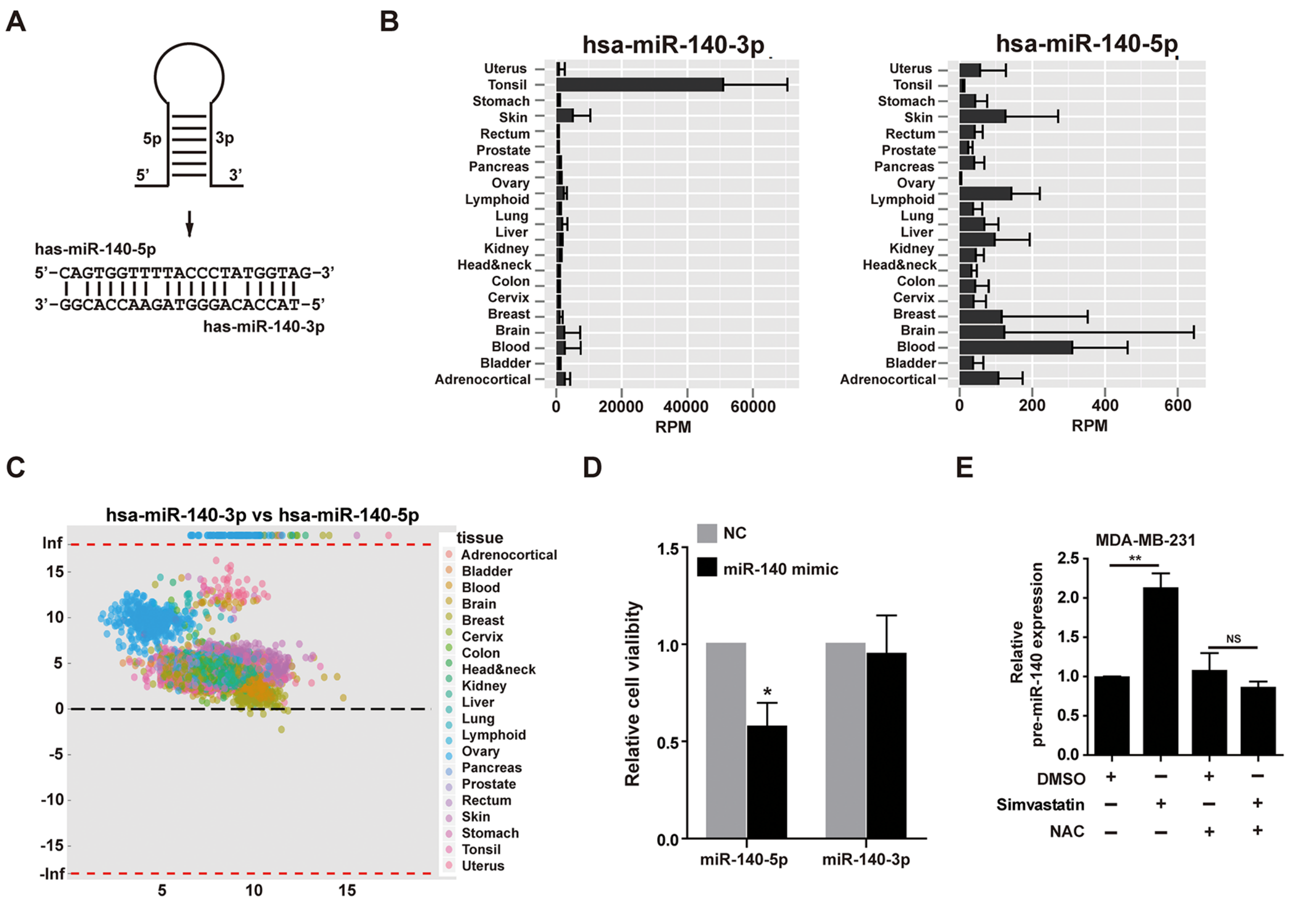

Figure S3. miR-140-3p is dominant in most human tissues compared with miR-140-5p. (**A**) The sequences of miR-140-3p and miR-140-5p. (**B**) Differential expression of miR-140-3p and miR-140-5p in human tissues, the data came from YM500 miRNA database. (**C**) Expression ratio of miR-140-3p vs miR-140-5p in human tissues, the data came from YM500 miRNA database. (**D**) MDA-MB-231 cells were transfected with 50nM miR-140-3p mimic or 50nM miR-140-5p mimic for 48h, NC as a negative control. Cell viability (cell proliferation) assays were analyzed by the CCK-8 assay. (**E**) The effect of simvastatin and NAC on pre-miR-140 expression as detected by qPCR.

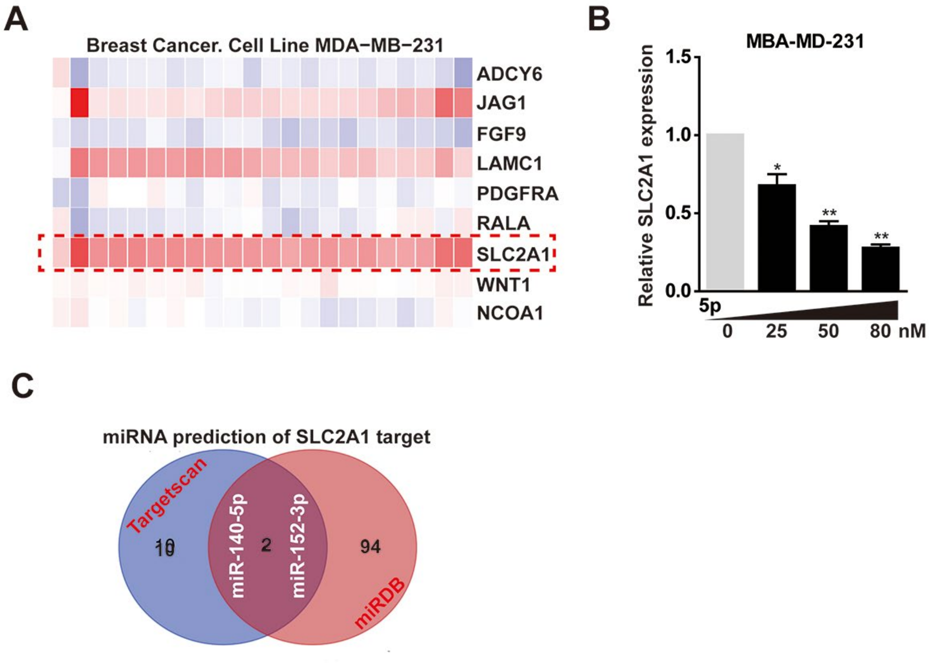

Figure S4. SLC2A1 is the downstream target gene of miR-140-5p. (**A**) The Heatmap showed the expression levels of the predicted targets of miR-140-5p in MDA-MB-231 cells and breast tumor by analyzing data from the MERVE database. (**B**) The expression levels of SLC2A1 was detected by qPCR in MDA-MB-231 cells transfected with varying concentrations of miR-140-5p mimic (0-80nM) for 48h. (**C**) Venn diagram containing the genes that were predicted to be the targets of miR-140-5p, miRNA prediction software targetscan and miRDB were used for this research.
